# The ventral epithelium of *Trichoplax adhaerens* deploys in distinct patterns cells that secrete digestive enzymes, mucus or diverse neuropeptides

**DOI:** 10.1242/bio.045674

**Published:** 2019-07-31

**Authors:** Tatiana D. Mayorova, Katherine Hammar, Christine A. Winters, Thomas S. Reese, Carolyn L. Smith

**Affiliations:** 1 Laboratory of Neurobiology, National Institute of Neurological Disorders and Stroke, National Institutes of Health, 49 Convent Drive, Bethesda, MD 20892, USA; 2 Central Microscopy Facility, Marine Biological Laboratory, 7 MBL Street, Woods Hole, MA 02543, USA; 3 Light Imaging Facility, National Institute of Neurological Disorders and Stroke, National Institutes of Health, 35 Convent Drive, Bethesda, MD 20892, USA

**Keywords:** Placozoa, Nervous system evolution, Digestive system evolution, Neuropeptide, Mucus, Gland cell

## Abstract

The disk-shaped millimeter-sized marine animal, *Trichoplax adhaerens*, is notable because of its small number of cell types and primitive mode of feeding. It glides on substrates propelled by beating cilia on its lower surface and periodically pauses to feed on underlying microorganisms, which it digests externally. Here, a combination of advanced electron and light microscopic techniques are used to take a closer look at its secretory cell types and their roles in locomotion and feeding. We identify digestive enzymes in lipophils, a cell type implicated in external digestion and distributed uniformly throughout the ventral epithelium except for a narrow zone near its edge. We find three morphologically distinct types of gland cell. The most prevalent contains and secretes mucus, which is shown to be involved in adhesion and gliding. Half of the mucocytes are arrayed in a tight row around the edge of the ventral epithelium while the rest are scattered further inside, in the region containing lipophils. The secretory granules in mucocytes at the edge label with an antibody against a neuropeptide that was reported to arrest ciliary beating during feeding. A second type of gland cell is arrayed in a narrow row just inside the row of mucocytes while a third is located more centrally. Our maps of the positions of the structurally distinct secretory cell types provide a foundation for further characterization of the multiple peptidergic cell types in *Trichoplax* and the microscopic techniques we introduce provide tools for carrying out these studies.

## Abstract (precis)

**Summary:** The external digestive epithelium of the primitive animal *Trichoplax adhaerens* includes secretory cell types and secretory products much like those in the internal digestive systems of other animals.

## INTRODUCTION

Placozoans are a group of early diverged multicellular marine animals characterized by a primitive cellular organization, which make them a promising model for understanding the early evolution of metazoan body plans and cell types. They are small, pancake-shaped animals that glide upon substrates, propelled by beating cilia. Their shapes are amorphous and continually changing (Grell and Benwitz, 1971; Grell and Ruthmann, 1991; Pearse and Voigt, 2007; Schulze, 1891a). They feed on microorganisms such as microalgae and cyanobacteria, which they digest externally in the narrow space between their lower surface and the substrate (Grell and Ruthmann, 1991; Smith and Reese, 2016). When left without food, they spend much of their time making random walks (Ueda et al., 1999). However, when food is present, they periodically cease moving and initiate behaviors involved in feeding (Smith et al., 2015; Ueda et al., 1999).

The ventral epithelium is pseudostratified and comprises several cell types that together account for 75% of the total cells in the animal (Smith et al., 2014). Most prevalent are the monociliated ventral epithelial cells (VEC; Ruthmann et al., 1986; Smith et al., 2014) whose cilia propel gliding (Grell and Ruthmann, 1991; Schulze, 1891b; Smith et al., 2015). Their cilia beat regularly, but asynchronously, and transiently contact the substrate during each stroke (Smith et al., 2015). The VEC readily phagocytose tracers added to the ambient seawater (Ruthmann et al., 1986; Smith and Reese, 2016) and therefore are thought to function in nutrient uptake. At the edge of the animal the ventral epithelium joins the dorsal epithelium, which is composed of the much broader apical terminations of dorsal epithelial cells. The nuclei of the dorsal cells protrude into the animal giving them a T-shaped appearance (Smith et al., 2014), similar to pinacocytes in the epithelia of Porifera (Bagby, 1970). Like pinacocytes, dorsal epithelial cells are contractile (Armon et al., 2018). All epithelial cells are joined by adherens junctions that fix their positions relative to each another (Smith and Reese, 2016). Neither occluding junctions nor gap junctions are present and there is no basal lamina (Grell and Ruthmann, 1991). In between the dorsal and ventral epithelia are fiber cells, which are reported to be contractile (Behrendt and Ruthmann, 1986; Buchholz and Ruthmann, 1995; Thiemann and Ruthmann, 1989), and infrequent crystal cells, which are functional statocysts (Mayorova et al., 2018).

Several types of secretory cells are included in the ventral epithelium. The most prevalent type is the lipophil cell, so called because of its content of large (up to 3 µm) lipophilic granules (Smith et al., 2014, 2015). In animals feeding on microalgae, a subset of the lipophil cells that are in the close vicinity (<15 µm) of an algal cell release their large apical granule, which rapidly is followed by lysis of the algae (Smith et al., 2015). While these observations point to lipophils as digestive cells, their digestive enzymes have not previously been identified, and it is not known how their secretory activity is regulated.

In addition to lipophils, the ventral epithelium contains gland cells, a generic term used to refer to cells containing numerous membrane-packaged granules smaller than those in lipophil cells. The granules in different cells uniformly vary in size and electron opacity, which suggests that *Trichoplax* possesses multiple types of gland cells (Grell and Ruthmann, 1991; Smith et al., 2014). The first evidence that some of the secretory cells might be peptidergic came from a light microscopic study (Schuchert, 1993) in which animals were immunolabeled with an antibody against a short synthetic peptide, RFamide, that reacts with many, although not all, peptides that have RFamide at their C terminus (Greenberg et al., 1988). The labeled cells were located in a narrow zone a short distance from the edge of the animal, as recently confirmed (Varoqueaux et al., 2018). Antibodies against FMRFamide (Smith et al., 2014) and endomorphin 2 (YPFFamide) (Senatore et al., 2017) label a row of cells at the edge of the ventral epithelium. The similarity in the distributions of cells labeled by anti- FMRFamide and endomorphin 2 has been attributed to cross-reactivity (Senatore et al., 2017).

Neuropeptides are synthesized in the endoplasmic reticulum and cleaved and processed in the Golgi complex (Fricker, 2008; Sossin et al., 1989). Many of them have a C-terminal glycine that is converted to an amide group by peptidyl-glycine-alpha-amidating monooxygenase. The presence of a C-terminal amide is thought to stabilize the peptide and usually is required for biological activity (Fricker, 2008; Sossin et al., 1989). No prepropeptide for an RFamide-like peptide has been found in *Trichoplax* (Nikitin, 2015). However, a prepropeptide found in *Trichoplax* transcriptome (Senatore et al., 2017) contains several repeats of an endomorphin 2-like sequence (QDYPFFGN/S) flanked by dibasic amino acids, the signals for cleavage of the prepropeptide, but the C-terminal asparagine/serine makes it uncertain whether this peptide is amidated.

Senatore and co-authors (2017) reported that applying >200 nM endomorphin 2 or QDYPFFamide to the bath around gliding *Trichoplax* reliably arrested ciliary beating and elicited a pause in movement similar in duration to that exhibited during feeding. By contrast, FMRFamide and the unamidated peptide, QDYPFFNG, elicited pausing only in ∼40% of animals and high concentrations of peptide were needed. The cells expressing an endomorphin-like peptide might be chemosensory cells that secrete peptide upon detection of algae so as to arrest movement of the animal while it feeds (Senatore et al., 2017).

Several additional peptides identified in the *Trichoplax* genome (FFNPamide, WPPF) elicit pausing when applied to the medium around moving animals (Varoqueaux et al., 2018), but whether they arrest ciliary beating remains to be determined. Additional peptides with distinct effects on *Trichoplax* behavior have been identified and the locations of some of them have been mapped by immunolabeling. Each labeled cell population has a distinct distribution (Varoqueaux et al., 2018), but none was located close to the edge of the ventral epithelium where cells labeled by anti-FRMR/YPFFamide reside.

Ciliated epithelia typically contain mucocytes that secrete mucus, a sticky substance containing highly glycosylated proteins. Other animals that, like *Trichoplax*, locomote by ciliary gliding require secreted mucus for adhesion and traction on the substrate (Martin, 1978; Wotton, 2004). Although it has been reported that *Trichoplax* secretes a sticky substance (Smith et al., 2015), mucus secreting cells have not previously been identified.

The purpose of the present study was to obtain a closer look at the secretory cell types in the ventral epithelium of *Trichoplax* and to learn more about their roles in locomotion and feeding. We employed serial section scanning electron microscopy (SEM) to identify, reconstruct and map the positions of the morphologically distinct secretory cell types. Transmission electron microscopy (TEM) provided a higher resolution picture of their structural features including their distinctive apical endings. Nanogold label allowed us to identify cells that react with anti-YPFFamide antibody and with a lectin that binds to mucus. Light microscopy of whole animals stained with fluorescent lectins provided a more quantitative map of mucocytes and fluorescence *in situ* hybridization (FISH) allowed us to localize digestive enzymes in lipophil cells. The role of mucus in locomotion was investigated by comparing the behavior of animals exhibiting normal and experimentally reduced rates of mucus secretion. We show here that *Trichoplax* deploys a variety of secretory cells in its ventral epithelium arranged in distinctive patterns appropriate to their roles in locomotion and feeding.

## RESULTS

### Types of secretory cell in the ventral epithelium

**Fig. 1.**
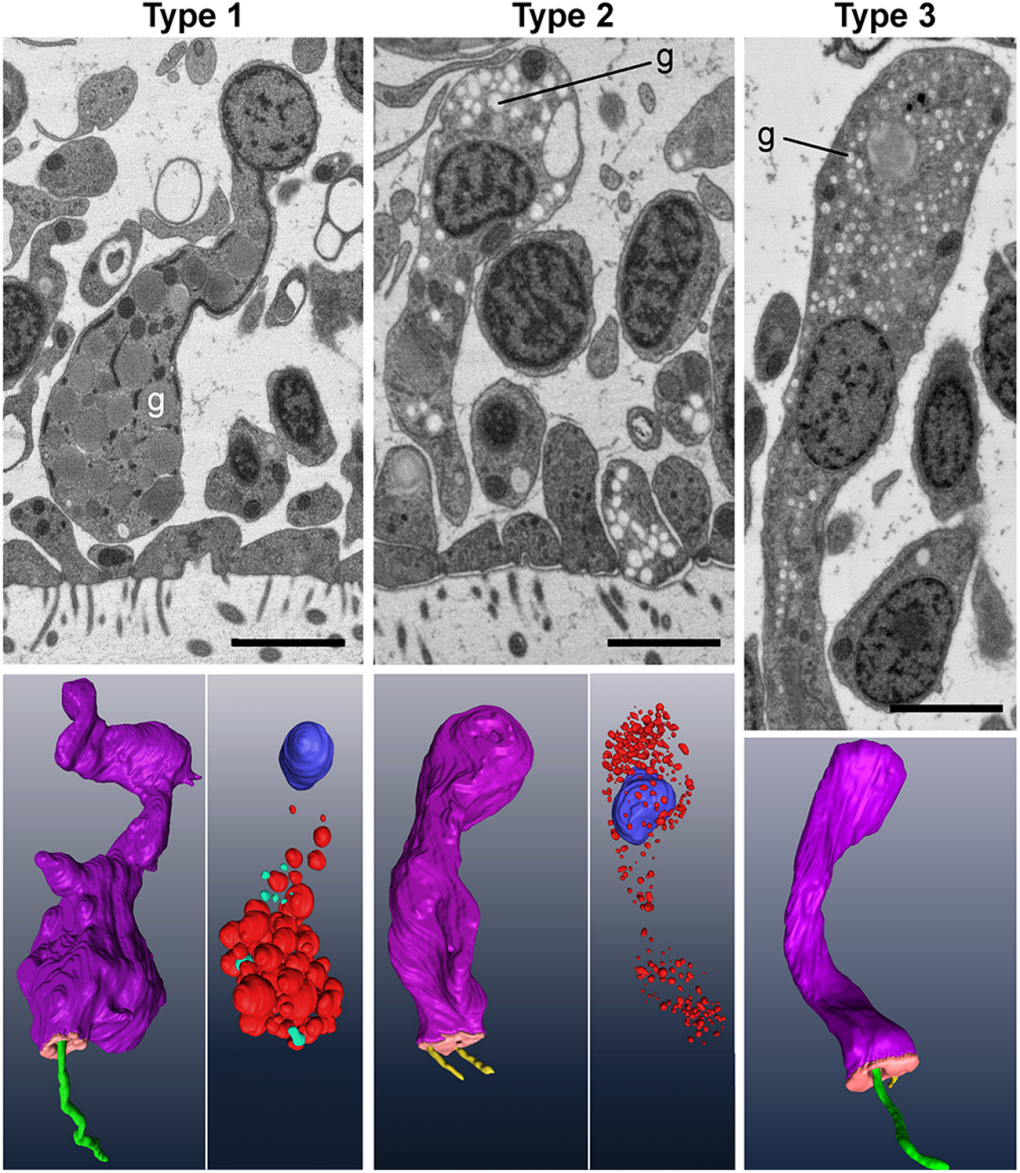
**Three structural types of gland cell in *Trichoplax* ventral epithelium.** Upper row shows gland cells in thin sections in SEM backscatter mode, and lower row shows three-dimensional renderings. Cell body, purple; apical surface, salmon; cilium, green; microvilli, yellow; nucleus, blue; internal granules, red. Gland cells are distinguished by their content of secretory granules (g). Type 1 cells have large granules with a dense content, while Type 2 and 3 cells have smaller and paler granules. Type 1 and 3 cells have an apical cilium. Type 2 cells lack a cilium but have microvilli. Scale bars: 2 µm.

Examination of thin sections in the ventral epithelium confirmed the presence of cells containing granules typical of gland cells, but the granules and other ultrastructural features differed between cells, suggesting that there could be several types of gland cell. We addressed this issue by adapting a serial section backscatter SEM technique used to collect hundreds of sections for brain connectomics at nanometer resolution (Helmstaedter, 2013; Shahidi et al., 2015). This approach permitted us to reconstruct and compare entire gland cells from freeze-substituted animals (Fig. 1.) Three distinct types of gland cell were apparent: Type 1 cells, which were filled with large electron dense granules and displayed a cilium (Fig. 1, left); Type 2 cells, with smaller electron lucent granules and lacking a cilium (Fig. 1, middle); and Type 3 cells, which were ciliated like Type 1 cells but contained much smaller and more electron lucent granules (Fig. 1, right).

**Fig. 2.**
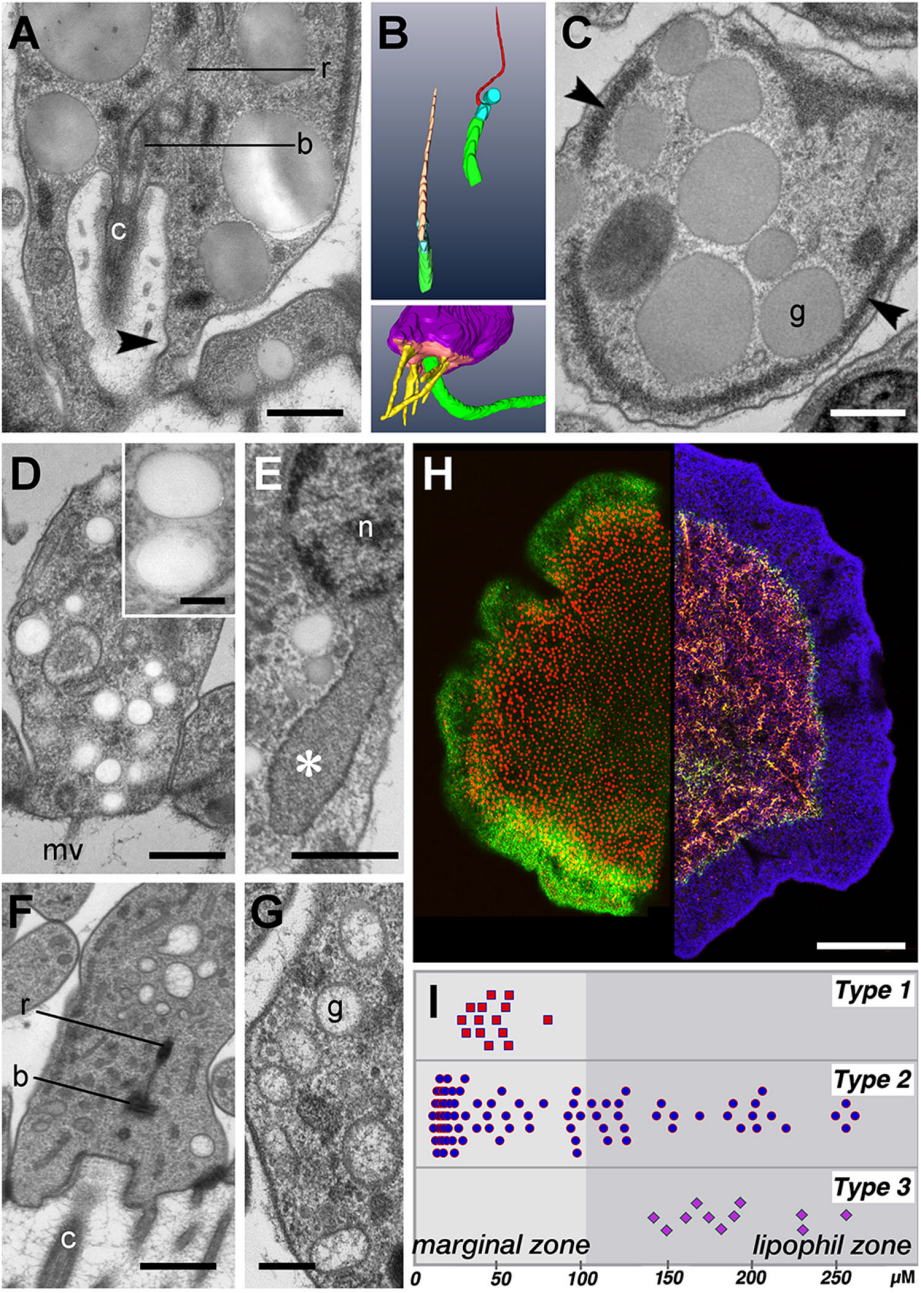
**Features and distributions of the three gland cell types and lipophil cells.** (A) Thin section from Type 1 gland cell shows its cilium (c) rising from a deep, ovoid pocket, the basal body (b) and the ciliary rootlet (r). Arrowhead indicates coated pit. (B) Three-dimensional reconstructions comparing the cilium and associated structures of a Type 1 gland cell and a VEC. Upper inset shows the short, thin ciliary rootlet (red, right) in a Type 1 cell and the longer and thicker rootlet in a VEC (orange, left), and associated basal bodies (cyan) and cilia (green). Surface renderings made from 12 serial sections. Lower inset shows the apical microvilli (yellow) surrounding the cilium (green) of a Type 1 cell. Surface rendering from 77 serial sections. (C) Thin section from the interior of a Type 1 cell shows distinctive large, grey granules (g) with homogeneous content and markedly dense ER (arrowheads). (D) Section from a Type 2 cell illustrates its clear granules and a microvillus (mv). Inset shows enlarged view of the clear granules. (E) Thin section of the area near the nucleus (n) of a Type 2 shows enlarged ER cistern (asterisk) characteristic of this cell type. (F) Thin section of the apical end of a Type 3 cell shows part of its cilium (c) arising from a shallow cup, the basal body (b) and a segment of the ciliary rootlet (r). (G) Enlarged view of the interior of a Type 3 cell shows the textured content of the granules. (H) Light micrographs showing two views of lipophil cells. Left half: lipophil granules in living animal stained with LipidTox (red). The surface of the animal is stained green with a lectin conjugated to a fluorescent dye (see below). Optical section at the ventral surface. Right half: FISH showing cells expressing phospholipase A (green) and trypsin (red). Nuclei are stained with Hoechst (blue). Maximum projection of a sequence of images comprising the entire animal. (I) Distribution of each type of gland cell mapped on a projection of half of the body of the animal. Map was created from tiled SEM images from one animal. Dark grey area indicates region containing lipophil cells. Scale bars: A,C,D–F: 500 nm; G: 200 nm; inset on D: 100 nm; H: 100 µm.

Once the distinguishing characteristics of the three types of gland cell were recognized, we examined each type in more detail in higher resolution TEM images where we could now be sure of the cell type. Type 1 cell cilia had a 9+2 organization of microtubules, as do cilia of VEC (Ruthmann et al., 1986; Smith et al., 2014). However, the ciliary pocket and basal apparatus of Type 1 cells differed from those of VEC – the ciliary pocket of the Type 1 cell was several times deeper than that of VECs and lacked supporting rods. In addition, the lining of the cup often contained one or more coated pits (Fig. 2A) while VEC ciliary pockets lack coated pits. The ciliary rootlet in Type 1 cells was thinner and shorter than that in VEC and was often curved (Fig. 2A,B, upper inset). Most of Type 1 cells had microvilli on their apical surface (Fig. 2B, lower inset), although microvilli were absent in some Type 1 gland cells (Fig. 1, left column), Type 1 cells had large (0.6–1.2 µm) secretory granules with homogenous content of medium (Fig. 2A,C) or, less frequently, dark electron density (Fig. 1 left, Fig. 2C). Another distinctive feature of Type 1 cells was their very electron dense endoplasmic reticulum (ER) (Fig. 2C).

Type 2 gland cells lacked a cilium but had multiple microvilli on their apical surface (Fig. 1 middle, Fig. 2D). Secretory granules were approximately 300 nm in diameter and had homogeneously electron lucent or pale contents (Fig. 2D). The granules were distributed throughout the cell body. Further differentiating Type 2 gland cells was their markedly enlarged ER, which formed wide cisterns near the plasma membrane and between organelles (Fig. 2E). Like other secretory cells, they had a well-developed Golgi apparatus.

Type 3 cells bore a single cilium and a few microvilli (Fig. 1 right column, Fig. 2F). The ciliary pocket was similar in depth to that of VEC and was framed with supporting rods (Fig. 2F). The ciliary rootlet was long and straight, as in VEC. Type 3 gland cells had the smallest granules among the three types of gland cell, with a diameter of ∼250 nm (Fig. 2F,G). The granule content that was textured, unlike that of Type 1 and Type 2 cell granules. Eleven of a total of 13 Type 3 gland cells were located in the ventral epithelium, but two were in the dorsal epithelium (Fig. S1).

Also included in the ventral epithelium were numerous lipophil cells with very large secretory granules. The ultrastructural features of lipophil cells have been previously reported (Smith et al., 2014, 2015).

### Radial distributions of secretory cells in the ventral epithelium

The outer edge of the animal is marked by VECs and a concentric zone containing lipophils begins approximately 100 µm farther in, leaving a zone between the edge of the animal and the lipophil zone that we refer to as the marginal zone (Fig. 2H; Smith et al., 2014). Though digestive activity of the lipophil granules was demonstrated previously (Smith et al., 2015), their content was not identified. Fluorescence *in situ* hybridization with probes against three digestive enzymes, trypsin, chymotrypsin and phospholipase A2, which are highly expressed in *Trichoplax* (Ringrose et al., 2013; Sebé-Pedrós et al., 2018; Wong, 2018), revealed that these enzymes are abundant throughout the zone that contains lipophils (Fig. 2H; Fig. S2A). FISH on dissociated cells confirmed that the cells expressing these enzymes are lipophils (Fig. S2B–E). Hence, the ventral epithelium is subdivided into two functional zones, a digestive (lipophil) central zone and non-digestive marginal zone.

The distributions of the three gland cell types were mapped from montages constructed from serial thin sections, in which cell types could be readily recognized and accurately positioned with respect to the marginal and lipophil zones. Type 2 cells, ∼65% of total gland cells, are most prevalent around the edge, in the outer 30 µm of the marginal zone (Fig. 2I; Fig. S3A–C). They are present in moderate numbers in more interior parts of the marginal zone and in the outer 100 µm of the lipophil zone. Their concentration declines with increasing distance into the lipophil zone and they are absent in the center of the animal. Type 1 cells, ∼20% of total gland cells, occur only in the narrow band within the marginal zone, beginning approximately 30 µm from the edge and extending ∼50 µm (Fig. 2I; Fig. S3A–C). Type 3 cells, the least abundant type (∼15% of total gland cells), are scattered in a 150 µm band within the outer part of the lipophil zone leaving both the marginal zone and much of the central lipophil zone free of Type 3 gland cells (Fig. 2i; Fig. S3B,C). The radial positions of Type 3 cells in the dorsal epithelium were similar to those of Type 3 cells in the ventral epithelium.

### Type 2 gland cells are mucocytes

**Fig. 3.**
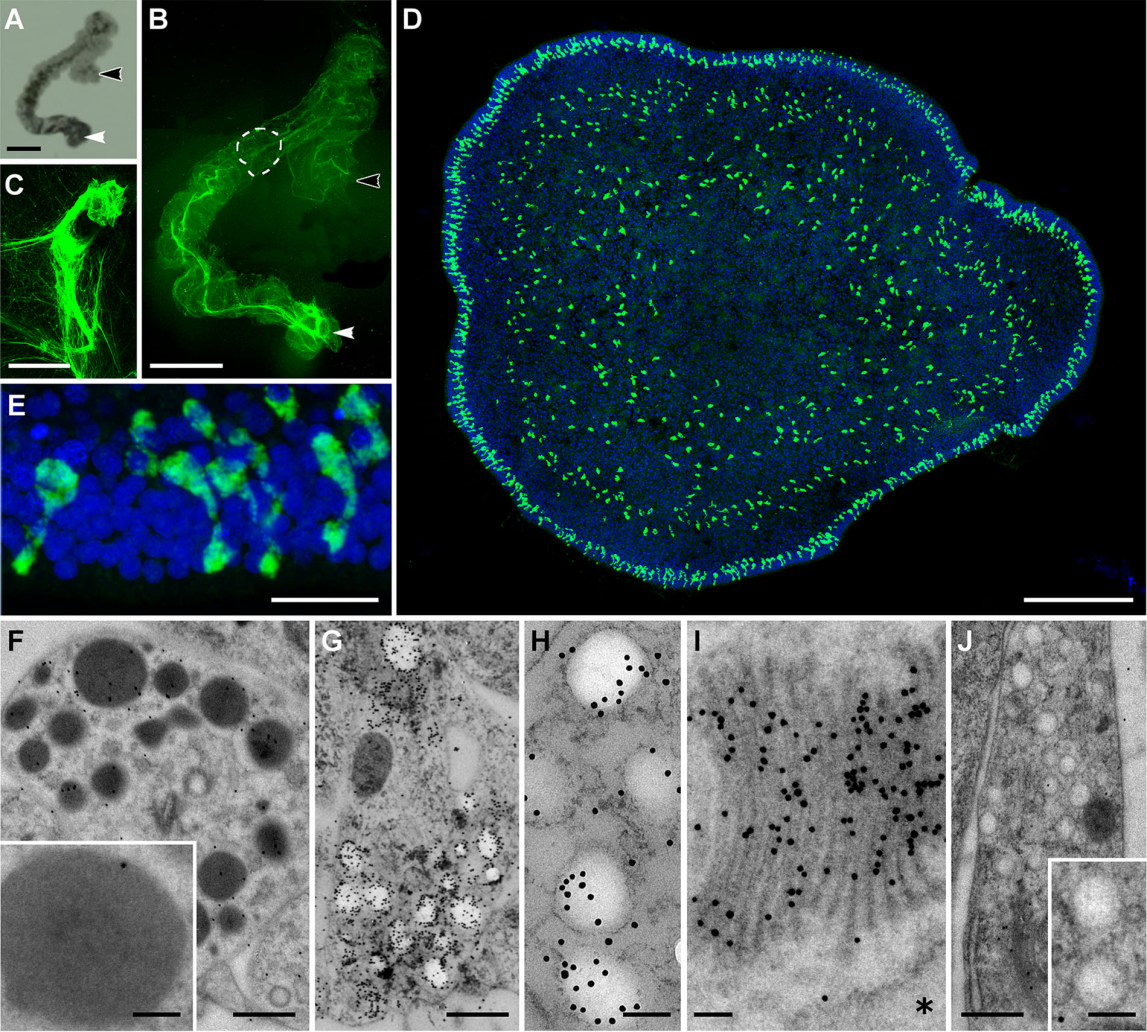
**Type 2 gland cells contain and secrete mucus.** (A) Projection of a time-lapse bright field image sequence (1.5 h) of a crawling animal. White arrowhead indicates start point and black arrowhead the end point. (B) Trail of mucus left by the crawling animal shown in A after staining with fluorescent WGA (green). Dotted line outlines the animal at one time point. (C) Enlarged view of the mucous clot left at the start point of the trail. (D) Mucus containing cells (mucocytes) labeled with WGA (green) in an *en face* view of an animal. The image is a maximum intensity projection of a series of optical sections through the animal. Mucocytes are numerous along the rim of the animal and present in moderate numbers further inside, but are absent near the center. (E) Enlarged view of WGA-stained mucocytes located near the rim of an animal. Projection of 38 optical sections. (F–J) TEM images of Types 1, 2 and 3 gland cells in thin sections labeled with WGA-nanogold. (F) Type 1 cell granules (overview and enlarged in inset). Few nanogold particles are present. (G) Numerous nanogold particles congregate in and around clear granules of Type 2 cells. (H) Enlarged view of Type 2 cell showing nanogold-WGA label in and around granules. (I) Type 2 cell Golgi complex labeled with WGA. Asterisk marks ER cistern. (J) Type 3 cell granules (overview and enlarged in inset). Few nanogold particles are present. Scale bars: A,B: 500 µm; C: 50 µm; D: 100 µm; E: 10 µm; F,G,J: 500 nm; H,I, insets on F,J: 100 nm.

As *Trichoplax* gliding on a substrate leave a trail of sticky substance along their track (Smith et al., 2015), we supposed that one of the gland cell types might secrete mucus. Mucins, gel-forming proteins that constitute one of the main components of mucus, are typically heavily glycosylated and therefore can be expected to bind to specific lectins. We screened a panel of fluorescent conjugated lectins (concanavalin A, peanut agglutinin, *Ricinus communis* agglutinin, soy bean agglutinin, *Ulex europaeus* agglutinin and wheat germ agglutinin) and discovered that one of them, wheat germ agglutinin (WGA), had a strong affinity for *Trichoplax* mucous trails. Peanut agglutinin faintly stained mucous trails (not shown), but no other lectin tested labeled them. A thin layer of WGA stained material was detected on both the lower (Fig. 2H) and upper surface of the animals (not illustrated). Gliding animals left a trail of mucus in their wake (Fig. 3A–C). Preabsorption of the lectin with the sugar moiety recognized by WGA (N-acetylglucosamine, GlcNAc) abolished the staining.

Staining wholemounts of animals with fluorescent WGA revealed a densely packed array of mucus containing cells (mucocytes) around the edge of the animal (Fig. 3D,E). The mucocytes were incorporated in the epithelium and had club-shaped apical endings (Fig. 3E). Interior to these cells was a narrow (∼30 µm) zone that was free of mucocytes (Fig. 3D). Modest numbers of mucocytes were present in the inner part of the marginal zone and outer part of the lipophil zone. The distribution of WGA stained mucocytes corresponded quite closely to that of Type 2 gland cells (Fig. 2I). Examination of thin sections labeled with WGA conjugated to 10 nm nanogold demonstrated that WGA nanogold accumulated on the electron lucent granules (Fig. 3G,H) and Golgi apparatus (Fig. 3I) of Type 2 cells. Pre-incubation with GlcNAc reduced WGA labelling seven times (∼100 versus ∼700 nanogold/µm^2^ over the Type 2 cell granules). Type 1 (Fig. 3F) and Type 3 (Fig. 3J) gland cells showed no significant staining above background with WGA.

### Mucus secretion supports adhesion and gliding locomotion

*Trichoplax* exhibit a series of stereotyped movements after detachment from the substrate and during reattachment. Upon detachment, they fold up with their densely ciliated ventral surface inside the fold. As they re-establish contact with the substrate, they remain folded but begin to rotate (Movie 1). Then the upper side of the fold begins to part from the lower side and establishes contact with the substrate. The zone of contact between the upper fold and the substrate expands and the animals gradually unfold. The process of reattachment and unfolding takes several minutes. After unfolding the animals exhibit varying rates of movement (10–20 µm/s in the absence of food; Ueda et al., 1999). Correlating the behavior of animals after placement on a glass coverslip with the pattern of WGA stained mucus observed afterwards showed that the animals leave a tangled mass of mucus at the site of initial attachment (Fig. 3B,C) and a thin meshwork of mucus and long mucous strands along their path of migration (Fig. 3A,B). The greater abundance of mucus at the site of attachment could reflect the longer time spent at that location.

*Trichoplax* in calcium free seawater (CF-ASW) are reported (Senatore et al., 2017) to have impaired gliding although their cilia continue to beat. Animals in CF-ASW attached to the substrate less firmly than animals in ASW, as evident from the ease with which they could be detached by a stream of liquid (not illustrated), and time lapse images showed that they migrated over much shorter distances (Fig. S4). WGA staining of their trails (Fig. S4) revealed that they left a clot of mucus at the site where they attached to the substrate, but the clot was not as large or intensely stained as that left by animals in normal seawater. Except at the site of attachment, they left very little mucus along their paths of movement (Fig. S4).

### Mucus secretion and feeding

**Fig. 4.**
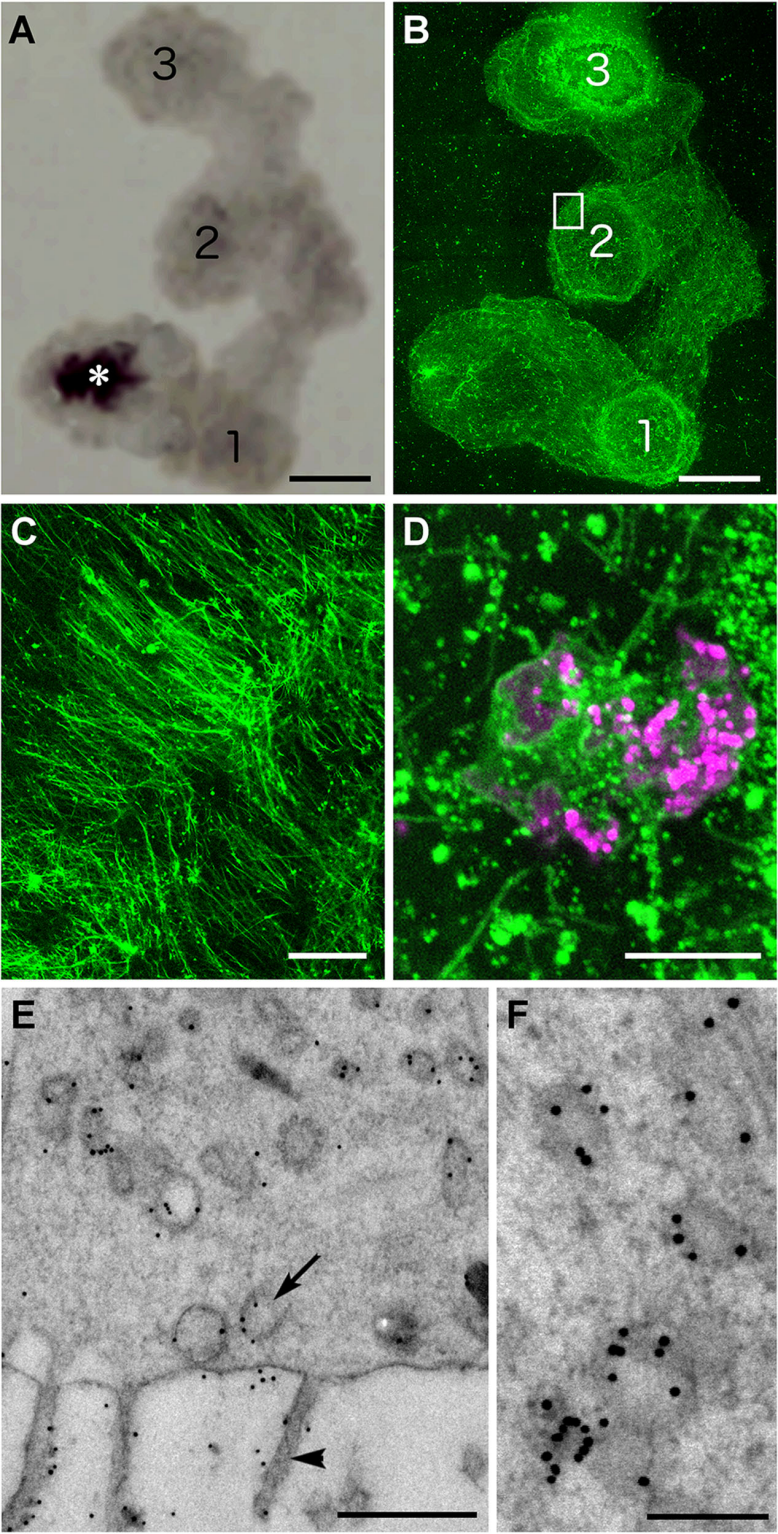
**Mucus secretion during feeding.** (A) Projection of a time-lapse bright field image sequence (40 min) of a starved animal (gray) placed on a lawn of algae (not visible). White asterisk indicates the start point. After attaching to the substrate, the animal began to crawl but periodically paused to feed. Sites of feeding pauses are labeled 1–3. (B) Mucous trail of the animal shown in A stained with fluorescent-WGA (green). (C) Higher magnification view of the area in the small box in B shows the orientations of mucous strands that accumulated around the edge of the animal during feeding. (D) Mucus surrounding debris from lysed algal cells (magenta) beneath an animal fixed during feeding. (E) Thin section of the apical part of a VEC labeled with nanogold-conjugated WGA. Nanogold particles coat the microvilli (arrowhead) and apical plasma membrane, and label phagosomes (arrow) in the interior. (F) Enlarged view of VEC phagosomes labeled with WGA-nanogold. Scale bars: A,B: 500 µm; C: 20 µm; D: 10 µm; E: 500 nm; F: 200 nm.

*Trichoplax* cease gliding for several minutes while feeding upon algae entrapped under their lower surface (Smith et al., 2015). WGA staining of trails of feeding animals revealed that places where the animal paused accumulated more fluorescent WGA than other parts of their trails and had a distinctive deposition of mucus (Fig. 4A,B). The area of mucus deposited during pauses was slightly larger than the diameter of the animal and brightly stained strands of mucus extended directly outward from the edge (Fig. 4C). The mean intensity in the zone at the edge was consistently greater (1.2- to 2.0-fold) than the intensity under the interior of the animal or along the paths of gliding animals. This difference in intensity could indicate that feeding is accompanied by enhanced mucus secretion at the edge of the animal. However, it also is possible that the rate of mucus secretion is unaltered during feeding and that the difference in intensity arises because the edge remains in the same place for longer during pauses. An argument against the second explanation is that the intensity of staining around the edge and underneath pausing animals was unrelated to the duration of the pause (Pearson coefficient −0.4). The rate of WGA binding to mucus (4 min to reach half maximal intensity) was too slow to allow us to use fluorescent WGA to monitor the time course of mucus secretion.

Animals that were fixed while they were pausing to feed had remnants of lysed algae entangled in the meshwork of mucous strands under their surfaces (Fig. 4D). Examination of WGA-nanogold stained thin sections of VEC, the cell type that is thought to take up nutrients during feeding, showed that their apical surfaces and microvilli were coated with mucus and that they had numerous mucus-containing vesicles in their interiors (Fig. 4E,F). These vesicles likely represent endocytic vesicles because they were similar in size and distribution to endocytic vesicles labeled by exogenous ferritin (Ruthmann et al., 1986) and some of them had coats (arrow in Fig. 4E).

### Endomorphin in gland cells

**Fig. 5.**
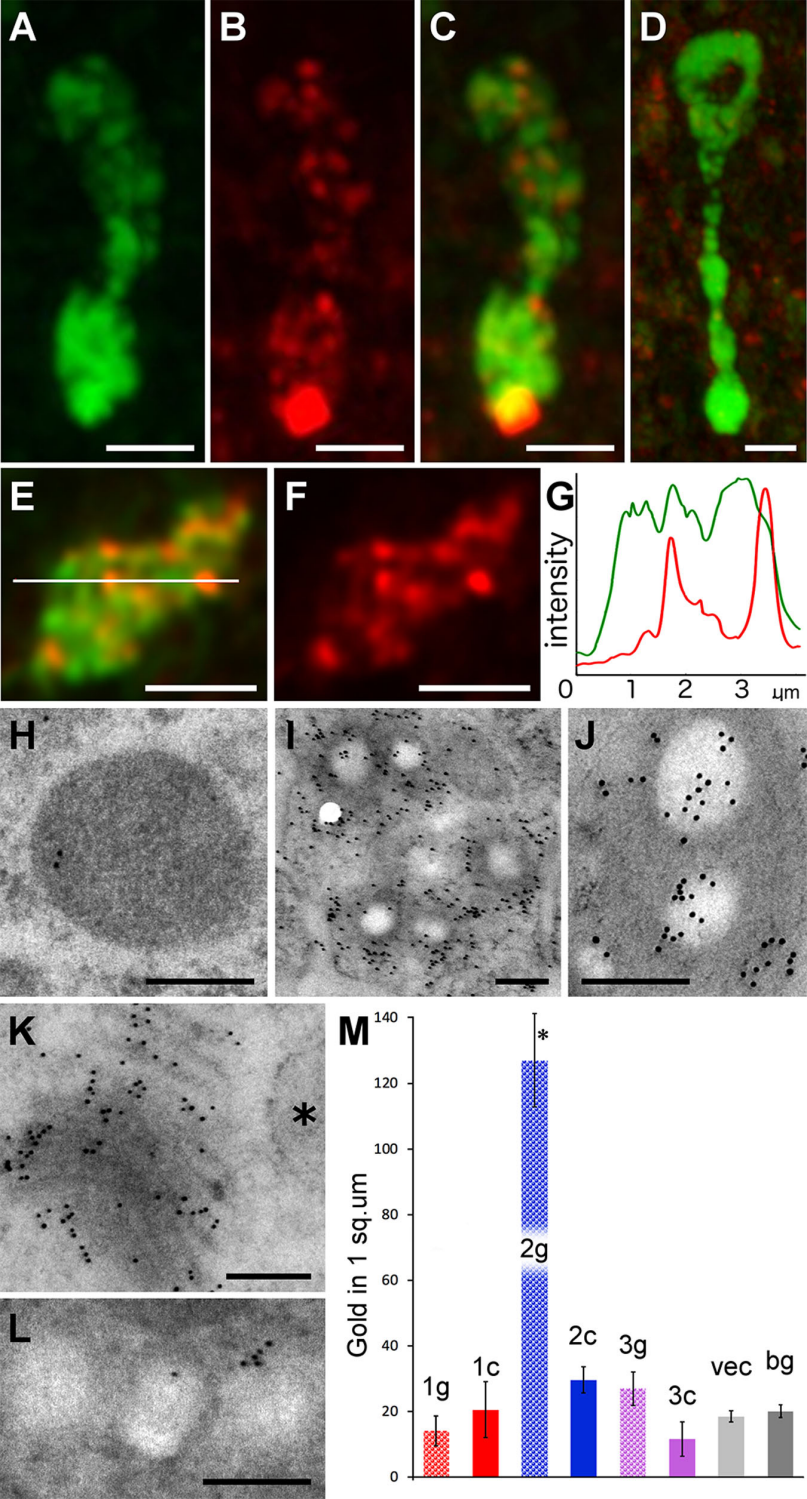
**Localization of mucus and endomorphin immunoreactivity in Type 2 gland cells.** (A–F) Confocal images from a whole animal stained with fluorescent WGA (green) and immunolabeled for endomorphin 2 (red). (A–C) Color separated and merged images of a Type 2 cell located at the edge of the animal. A cluster of granules near the apex and some granules deeper inside label with both WGA and anti-endomorphin 2. Projections of 25 optical sections. (D) A Type 2 gland cell farther from the edge shows no immunoreactivity for endomorphin 2. (E,F) Single optical section through the middle of a Type 2 gland cell body (E, merged; F, red channel). (G) Profile of green and red channel fluorescence intensity along the white line in E shows varying levels of endomorphin 2 immunoreactivity in WGA stained granules. (H–L) TEM images of thin sections labeled with anti-endomorphin 2 and immunogold-conjugated secondary antibody. (H) Granule in Type 1 cell shows no more than background immunogold labeling. (I) Type 2 cells demonstrate endomorphin immunoreactivity over and surrounding their clear granules. (J) Higher magnification immunolabeled Type 2 gland cell granules. (K) Endomorphin 2 immunogold labeling in the Golgi complex of a Type 2 cell. ER cistern (asterisk) is not labeled. (L) Granules in Type 3 cell show no more than background immunoreactivity. (M) Graph of the density (mean±s.e.m.) of endomorphin-nanogold in three gland cell types (1–3), with grains over granules (g) and cytoplasm (c) shown separately, compared to VEC and background (bg). Nanogold density over Type 2 cell granules (asterisk) is significantly higher than over cytoplasm or granules in the other cell types. Scale bars: A–F: 2 µm; H–L: 200 nm.

The subset of mucocytes arrayed around the edge of *Trichoplax* had similar shapes and distributions to cells that immunolabel for endomorphin 2/FMRFamide (Senatore et al., 2017; Smith et al., 2014). Double staining for mucus and endomorphin 2 showed that a large fraction of mucocytes around the edge immunolabeled for endomorphin 2 (Fig. 5A–C), but only a few of the mucocytes farther in the interior were labeled (Fig. 5D). The endomorphin 2 staining was most concentrated at the apical ends of the cells whereas the mucus staining was more uniformly distributed throughout the cell (Fig. 5A–C). The mucus staining partially overlapped the endomorphin 2 staining (Fig. 5C,E,F). Analyses of line intensity profiles confirmed that some WGA labeled structures were also labeled for endomorphin 2 (Fig. 5G). Colocalization analysis (not shown) yielded low cross-correlation values, consistent with partial overlap of the signals.

Post-embedding staining of ultra-thin sections with anti-endomorphin was used to determine the intracellular localization of the epitope. Since post-embedding immunolabeling typically generates some background, quantitative analysis of the distribution of label was performed by counting gold particles within circular 400 nm diameter masks superimposed over granules and cytoplasm in gland cells and, for comparison, over VEC and intercellular spaces (see Materials and Methods).

Most Type 2 gland cells had significant labeling for endomorphin on and around some of their granules (Fig. 5I,J). However, the density of labeling differed markedly between granules, with 41 of 56 showing a density of nanogold up to 20-fold higher than background and 15 showing only the background density. Four of 18 Type 2 gland cells had no immunogold labeling in their interiors. Nevertheless, the average density of nanogold labeling for endomorphin in all Type 2 cell granules [127.0±106.1 (s.d.) nanogold/µm^2^, *N*=56 sample areas, *P*<0.001, ANOVA], including those with no label, was significantly higher than background [20.0±15.0 (s.d.) nanogold/µm^2^, *N*=60 sample areas; Fig. 5M]. The Type 2 cells that contained nanogold-labeled granules also had label on their Golgi apparatus (Fig. 5K). No significant labeling was present in Type 1 or Type 3 gland cells or in VEC (Fig. 5H,L,M).

**Fig. 6.**
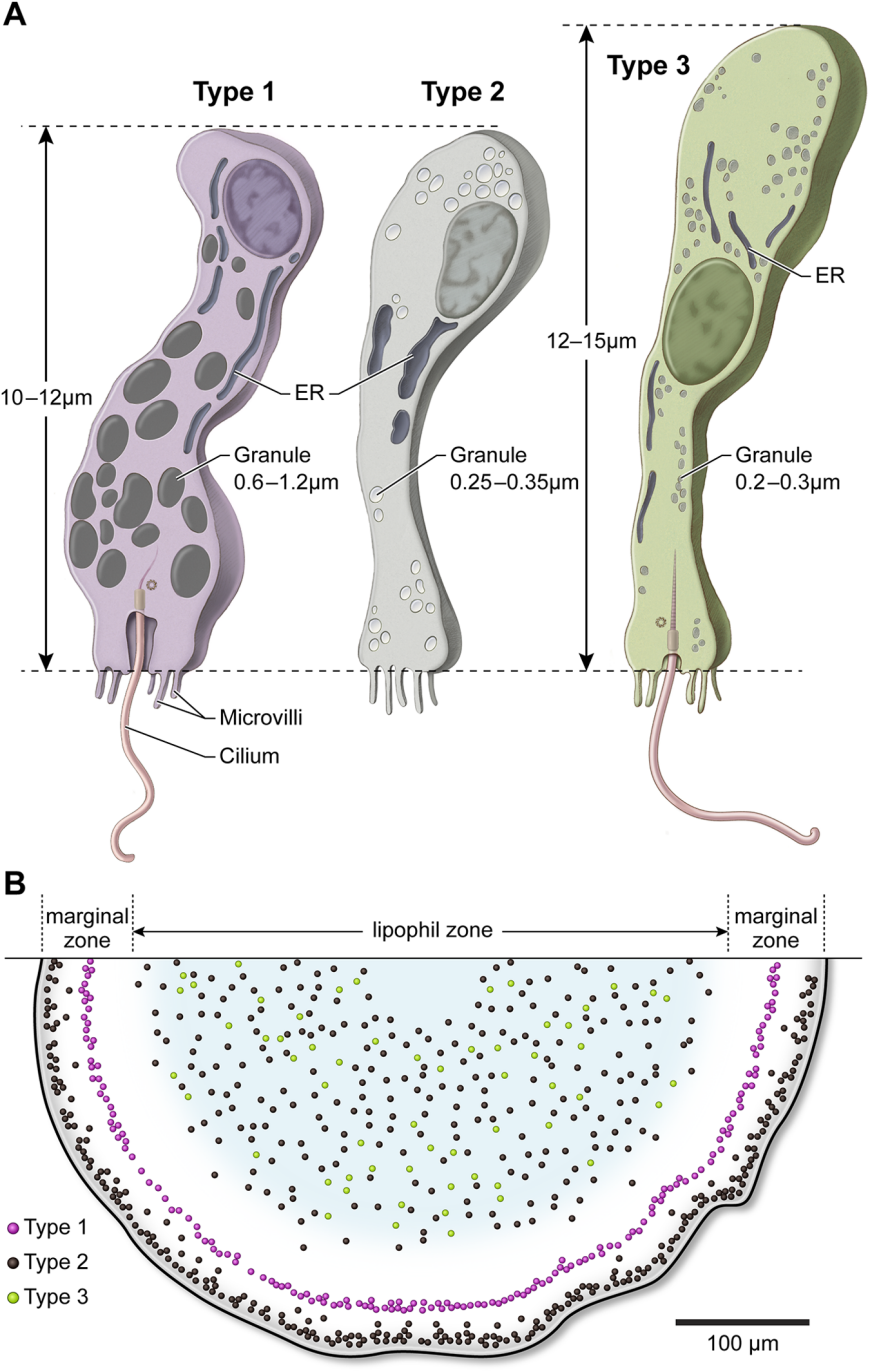
**Diagrams showing three gland cell types and their distributions in the ventral epithelium.** (A) Type 1 cells are distinguished by large, electron dense secretory granules and a single cilium arising from a deep pit. Type 2 gland cells lack a cilium and have smaller, secretory granules with electron lucent content. Type 3 gland cells have a single cilium and the smallest secretory granules with a textured content. (B) Map of gland cell positions. Type 2 cells are nearest to the edge while Type 1 cells occupy a separate concentric ring further inside. Type 2 and Type 3 cells are intermingled in the lipophil zone but are absent in the center.

The morphology, distribution, and some chemical signatures of the three types of gland cells found in the ventral epithelium of *Trichoplax* are summarized in Table 1 and Fig. 6 (A,B).

**Table 1.**
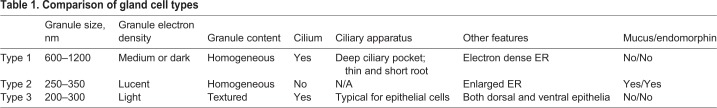
**Comparison of gland cell types**

## DISCUSSION

The ventral epithelium of *Trichoplax* combines two vital functions – locomotion and digestion. Electron microscopy provided a closer look at the different types of secretory cells and their locations in the ventral epithelium. Serial SEM allowed us to reconstruct whole secretory cells, revealing their specialized architectures, and to map the distributions of both the gland cells and the lipophils, which secrete digestive enzymes. Contents of secretory granules were identified by immunogold TEM and correlated with maps obtained from staining whole animals with fluorescent labels for secreted peptides and proteins. Here we discuss what has been learned about the secretory cell types, their secretory products and their roles in locomotion and digestion.

Lipophil cells, the most prevalent secretory cell type (Smith et al., 2014), secrete granules whose contents lyse algae (Smith et al., 2015). Here we show by FISH that lipophil cells express trypsin and chymotrypsin, enzymes that are widely used in eukaryotic cells for intracellular and extracellular digestion (Muhlia-Almazán et al., 2008), and a homolog of the pancreatic type of phospholipase A2 (Wong, 2018). As lipophils apparently are the only cells that express high levels of these enzymes, it is likely that the enzymes are constituents of their secretory granules and are used for extracellular digestion.

The most prevalent type of gland cell is the Type 2 cell that contains small, clear granules that label with probes conjugated to WGA lectin. WGA binds to N-acetylglucosamine and sialylated glycoconjugates, carbohydrates common in mucins. The *Trichoplax* genome includes a sequence for a mucin-like protein with vWFD, C8 and Til domains characteristic of vertebrate gel-forming mucins (Lang et al., 2016), consistent with the possibility that *Trichoplax* mucus contains mucins. Staining whole animals with fluorescent WGA revealed that animals leave a mucous trail behind them as they glide on the substrate. Mucus secretion appears to be calcium dependent because it was much reduced in low calcium seawater. Moreover, gliding was impaired in calcium free seawater, suggesting that gliding in *Trichoplax* requires mucus for adhesion and traction as does ciliary gliding in other animals (Martin, 1978; Wotton, 2004).

Many granules in Type 2 cells are labeled by anti-endomorphin 2 (YPFFamide) and may be assumed to package an endomorphin 2-like-peptide (ELP). However, there is a need for caution because antibodies against peptides may cross-react (Conzelmann and Jékely, 2012; Greenberg et al., 1988). An antibody against a *Trichoplax* prepropeptide (TaELP) that contains QDYPFFGN/S repeats, also labels Type 2 gland cells (Senatore et al., 2017), further support for the conclusion that Type 2 cells package an ELP. The presence in *Trichoplax* of a cell type that expresses TaELP also has been confirmed by single cell RNA sequencing (Sebé-Pedrós et al., 2018).

The cells that label for endomorphin 2 and TaELP express voltage-gated calcium channels as well as proteins implicated in calcium dependent secretion (Senatore et al., 2017; Smith et al., 2014). Senatore and co-authors (2017) hypothesized that ELP-expressing cells may be chemosensory and secrete peptide upon activation by a chemical signal emitted from algae. In this respect, they parallel chemosensory enteroendocrine cells in intestinal epithelium of insects (Hartenstein, 2006) and vertebrates (Gribble and Reimann, 2016) that secrete paracrine signals that regulate motility of the gut. Type 2 cells have microvilli on their surfaces that could serve as chemosensors (Elsaesser and Paysan, 2007) for algae. Type 1 and Type 3 cells bear a cilium, which also often has a sensory function (Bloodgood, 2010), suggesting that these cell types might be additional examples of sensory secretory cells.

Although almost all clear granules in Type 2 cells label for mucus, only a subset label for endomorphin 2 and no structural differences between granules served to divide them into classes. Co-existence of two types of secretory granules is common in neurons and endocrine cells (Hartenstein, 2006; Rindi et al., 2004), although the granules typically differ in size and appearance. Many cells that exhibit regulated secretion also engage in constitutive secretion and some cells have two or more separate pathways for regulated secretion (De Camilli and Jahn, 1990).

The uniform distribution of mucus along the tracks of gliding animals suggests that mucus secretion is constitutive. However, secretion from granules containing an ELP is likely to be regulated and activated by a signal emitted by algae (Senatore et al., 2017). Since the granules labeled by anti-endomorphin 2 also contain mucus, secretion of peptide should be accompanied by mucus secretion. Enhanced mucus secretion prior to feeding could explain the accumulation of a ring of mucous strands around feeding animals. The mucous ring could help to confine digested material underneath the animal (Smith and Reese, 2016) by promoting adhesion and providing a barrier to diffusion. The presence of mucus in VEC phagosomes suggests that mucus also serves as a vehicle for uptake of food particles.

*Trichoplax* has genes for at least twelve predicted secretory peptides (Jekely, 2013; Nikitin, 2015; Varoqueaux et al., 2018; Wong, 2018) and synthetic versions of several peptides modulate its behavior (Senatore et al., 2017; Varoqueaux et al., 2018). The locations of cells that express six peptides have been mapped by immunofluorescence (Varoqueaux et al., 2018). Each cell type has a distinctive distribution, making it possible to compare their locations with those of the three gland cell types described here. The cells expressing insulin-like peptide (Ins3) are positioned similarly to Type 1 cells. FFNPamide- and YYamide-expressing cells are located in the lipophil zone and could correspond to Type 3 or centrally located Type 2 cells. The locations of cells labeled by other antibodies (Varoqueaux et al., 2018) do not correspond with any of our morphological secretory cell types. Additional morphological types of gland cell may await discovery.

The distinctive radial distributions of the different secretory cell types in *Trichoplax* ventral epithelium appear well suited to their proposed roles during feeding. Type 2 cells arrayed around the edge are positioned to secrete mucus needed to support gliding and to corral food particles during feeding, as well as to signal imminent encounters with algae. If, as has been proposed (Senatore et al., 2017), Type 2 cells bear receptors for the ELP that they secrete, then even a small number of cells activated by a signal emanating from algae could initiate a wave of ELP secretion extending across the entire animal so as to arrest beating of VEC cilia. The close packing of Type 2 cells around the edge might facilitate paracrine signaling between these cells. The abundance and uniform distribution of lipophil cells in the lipophil zone ensures that any algal cell under the animal will be near a lipophil cell. It remains a puzzle how lipophil secretion is regulated so as to ensure that lipophils close to algae secrete digestive enzymes whereas lipophils distant from algae do not (Smith et al., 2015). The sparse numbers and patchy distributions of gland cells and peptidergic cells (Varoqueaux et al., 2018) in the lipophil zone makes it unlikely that they have a role in the precise targeting of lipophil cell secretion.

The cell types in *Trichoplax* ventral epithelium bear structural and functional resemblances to cell types in digestive epithelia of Cnidaria and Bilateria. The absorptive cells, VEC, are structurally similar to ciliated enterocytes, the absorptive cell type in Cnidaria and many Bilateria (Harrison and Westfall, 1991; Takashima et al., 2013). Enterocyte cilia typically are motile, like VEC cilia, but their beating propels fluid flow rather than supporting locomotion. Lipophil cells express digestive enzymes present in exocrine gland cells in the pharynx of Cnidaria (Raz-Bahat et al., 2017; Steinmetz et al., 2017) and in parts of the digestive tracts of Bilateria (Karasov and Douglas, 2013). Type 1 gland cells may correspond to insulinergic enteroendocrine cells (Conlon et al., 2008; Ebberink et al., 1989; Falkmer, 1972; Falkmer et al., 1973; Steinmetz et al., 2017), which have been implicated in regulating many physiological processes including development, growth and metabolism (Bradshaw, 1982; Dodson and Whittingham, 2005; Dupont and Holzenberger, 2003). Type 2 gland cells are functionally analogous to mucocytes/goblet cells in that they contain and secrete mucus (Harrison and Westfall, 1991; Takashima et al., 2013). However, it appears that they also secrete a regulatory peptide and, in this respect, resemble enteroendocrine cells. Mucocytes and enteroendocrine cells in bilaterians are the progeny of the same stem cells and their differentiation is controlled by the same regulatory pathways (Barker et al., 2007; Yang et al., 2001). Their shared regulatory pathways suggest that the two cell types may be derived from an ancestral cell type that, like Type 2 cells, may have secreted both mucus and a regulatory peptide.

Phylogenetic trees for Metazoa based on comparison of sequenced genomes place Ctenophora or Porifera as the oldest animal phylum and Placozoa as sister to Cnidaria and Bilateria (Moroz and Kohn, 2016; Moroz et al., 2014; Ryan et al., 2010; Simion et al., 2017). Most Porifera do not digest food extracellularly but instead capture food particles for intracellular digestion (Godefroy et al., 2019; Leys and Eerkes-Medrano, 2006) using cells with a filter apparatus similar to that of choanoflagellates (Dayel and King, 2014), the eukaryotic cells that are the closest sister to Metazoa (Brunet and King, 2017; Cavalier-Smith, 2017; King et al., 2008; Richter and King, 2013). Ctenophora have an internal digestive system that includes absorptive cells bearing microvilli and infrequent cilia, mucocytes and several types of exocrine cells (Bumann and Puls, 1997; Hernandez-Nicaise, 1991). Their genomes include sequences for a mucin-like protein (Lang et al., 2016), trypsin-like serine proteases and secretory peptides (Moroz et al., 2014), but the morphological cell types that express these secretory products have not been determined. Further work is needed to determine whether digestive epithelial cell types in Ctenophora, Placozoa, Cnidaria and Bilateria are homologs.

Several authors have proposed that the animals with internal digestion systems may descend from a flat animal that fed by digesting food on its exterior surface (Arendt et al., 2015; Sperling and Vinther, 2010; Syed and Schierwater, 2002). If this hypothetical ancestor possessed the cell types and body plan of *Trichoplax*, it could evolve into a cnidarian-like polyp by invaginating to adopt the shape of a sac (Schierwater et al., 2009) with its digestive epithelium in the interior and ciliated cells, mucocytes and peptidergic gland cells concentrated around the opening. Indeed, *Trichoplax* temporarily acquires the shape of a sac when feeding on a large clump of algae, with its margin closely apposed to the substrate and its digestive epithelium enclosing the food inside (see Fig. 6D in Smith and Reese, 2016). The arrangement of cells in this sac-shaped *Trichoplax* resembles that of cnidarian polyps, which have ciliated cells, sensory secretory cells, and mucocytes in their mouths and the same cell types plus digestive cells in their gastroderm (Goldberg, 2002; Harrison and Westfall, 1991; Haynes, 1973; Raikova, 1995; Raz-Bahat et al., 2017; Steinmetz et al., 2017). Recent analyses of fossil ctenophores provide strong evidence that Ctenophora descended from animals with body plans similar to Cnidaria polyps (Zhao et al., 2019). As Placozoa are the only extant animals that digest food on their outer surface, they provide a unique opportunity to learn about the cell types and behavioral repertoires needed to effectively employ this primitive mode of feeding.

## MATERIALS AND METHODS

### Trichoplax culture

*Trichoplax adhaerens* (Schultze, 1883) of the Grell (1971) strain, a gift from Leo Buss (Yale University), were maintained in Petri dishes with artificial seawater (ASW; Instant Ocean, Blacksburg, VA, USA) containing 1% Micro Algae Grow (Florida Aqua Farms, Dade City, FL, USA) and red algae (*Rhodomonas salina*, Provasoli-Guillard National Center for Culture of Marine Plankton, East Boothbay, ME, USA), as described previously (Selvan et al., 2015).

### Electron microscopy

Animals were frozen at high pressure in a Baltec 010 (TechnoTrade, Manchester, NH, USA) freezing machine, freeze substituted and embedded in Epon as described previously (Mayorova et al., 2018; Smith et al., 2014).

Scanning electron microscopy with subsequent serial image processing (alignment, segmentation, rendering) was performed as described previously (Mayorova et al., 2018). Briefly, freeze-substituted animals embedded in Epon blocks were sectioned at ∼70 nm and collected on a continuous feed of Kapton tape using an ATUMtome tape collecting ultramicrotome (RMC Boeckleler, Tucson, AZ, USA). Tape with adhering sections was cut into strips and affixed to a 10 cm silicon wafer using double adhesive carbon tape. Each wafer containing about 200 sections on Kapton tape was carbon coated using a Denton vacuum DV-502 evaporator (Moorestown, NJ, USA) and imaged in a Zeiss SMT Supra 40VP SEM (Cambridge, UK) at 7KV using a Zeiss 4QBSD detector (Munich, Germany) and ATLAS 5 software version 5.1 (Carl Zeiss Microscopy GmbH Fibics Incorporated). Cell membranes and internal organelles were demarcated clearly so particular cells could be followed from section to section to achieve one complete series of each of the three gland cell types with many more corroborations from partial series.

Gland cell distributions were mapped in tiled images (60×60 µm square fields, 10 nm resolution) of seven cross-sections through the whole body of three animals, each section passing near the center and spaced ∼10 µm apart so as to avoid duplicate counts of the same cells. Animals were approximately 600 µm in diameter. From the tiled images, we measured the distance between each gland cell and the edge of the animal, defined as the place where the columnar ventral epithelium meets the thinner dorsal epithelium.

For post-embedding immunogold staining, animals were freeze substituted and embedded in Lowicryl HM20 resin (EMS, Hatfield, PA, USA) in a Leica EM AFS2 9 (Leica Microsystems, Vienna, Austria) by a procedure modified from Petralia and Wenthold (1999). Briefly, all samples were placed under liquid nitrogen in a vial with previously frozen 1.5% uranyl acetate (UA, Polyscience, Warrington, PA, USA) in methanol (Sigma-Aldrich, St Louis, MO, USA) at −90°C for 32 h and ramped to −45°C in 4° per hour steps. At −45°C samples were rinsed three times in pre-chilled methanol and infiltrated in graded concentrations of HM20 in methanol (1:1 for 2 h, 2:1 for 2 h, 100% HM20 for 1 h, and 100% HM20 overnight) at −45°C. Finally, samples were transferred to fresh 100% HM20 and irradiated with UV light during the following steps: held at −45°C for 24 h, ramped to 0°C with 5° per hour step, and held for 40 h. Embedded samples were then trimmed into blocks and 70 nm thick sections were collected on formvar, carbon coated 200 nm mesh gold grids (EMS, Hatfield, PA, USA). Some sections were used for post-embedding nanogold labeling, see below.

Both Epon and HM20 ultrathin sections were examined at 120 KV in a JEOL JEM 200-CX (Tokyo, Japan) transmission electron microscope and photographed with an AMT camera mounted below the column.

### Post-embedding labeling

Mucus labeling was performed with wheat germ agglutinin (WGA) conjugated with 10 nm gold nanoparticles (EY Laboratories, San Mateo, CA, USA). Thin sections on gold grids were treated with calcium and magnesium-free phosphate buffer (DPBS, pH 7.4; Mediatech, Herndon, VA, USA) for 5–10 min then left on a drop of WGA-nanogold diluted 1:50 in DPBS for 45–60 min before being rinsed four times in distilled water, blotted, air dried and counterstained with UA (Ted Pella, Redding, CA, USA) for examination by TEM. To control for non-specific binding, sections were treated with WGA-nanogold pre-incubated with 0.5 M N-acetylglucosamine (GlcNAc; Vector Laboratories, Burlingame, CA, USA) on a shaker for 1 h at room temperature.

Post-embedding immunocytochemistry (IC) was done according to the protocol of Petralia and Wenthold (1999). First, a series of dilutions of anti-endomorphin antibody (#H-044-11; Phoenix Pharmaceuticals, Burlingame, CA, USA) was tested to determine the concentration (1:320) that gave adequate labeling at the lowest background. Post-embedding staining of thin sections was performed on two animals at this concentration with identical results. Sections were rehydrated in phosphate buffered saline (PBS, pH 7.4; Crystalgen, Commack, NY, USA) for 20 min and then incubated for 60 min in blocking buffer (BB) consisting of 5% normal goat serum and 1% bovine serum albumin (Sigma-Aldrich, St Louis, MO, USA) in PBS. Sections were incubated with primary rabbit anti-endomorphin antibody diluted in BB overnight in a humid chamber at 4°C. After four rinses with 1% BSA in PBS, sections were treated with secondary goat anti-rabbit antibody conjugated with 10 nm gold particles (Ted Pella, Redding, CA, USA) diluted 1:20 for 3 h at room temperature. Grids were then rinsed four times with PBS and four times with distilled water, blotted, air dried, counterstained with UA and examined in a TEM. Controls performed without primary antibody incubation showed no significant labeling (<0.1 nanogold/µm^2^).

To evaluate anti-endomorphin labeling in different cells, random sampling was done by superimposing masks 400 nm in diameter over micrographs of gland cells. The mask diameter was chosen so as to be larger than the smallest granules but smaller than the largest granules. Masks were placed on low magnification images where cells and organelles could be seen clearly but gold particles were not visible. Masks were centered over cytoplasm, granules or intercellular space. The area under the mask was cut and saved in a separate file for further evaluation at a higher magnification. Nanogold particles within each mask were counted by hand and densities calculated as particles per µm^2^. ANOVA test (PAST 3.14 software) was used to measure significance of difference between counts on cells and background.

### Visualizing animal movements and mucous trails

Acid-cleaned glass coverslips were pretreated with amino silane prior to use to promote adhesion of the animals during subsequent fixation and processing (Smith et al., 2014). In some experiments, the coverslips were kept in a dish containing algae for ∼1 week so that they became coated with algae. The coverslip was placed in a petri dish or imaging chamber and flooded with ASW or calcium-free ASW made as described previously (Tamm and Tamm, 1981) except it was supplemented with 2 mM EGTA (Sigma-Aldrich, St Louis, MO, USA). Time-lapse photography was performed with a Canon EOS 5D Mark III 22.3 MP digital camera equipped with Canon MP-E 65 mm f/2.8 1-5x Macro Photo lens (Canon, Melville, NY, USA) as described previously (Mayorova et al., 2018). Observations were carried out in a darkened room at a constant temperature. Recordings were started immediately after the animal was placed in the dish. Images were taken every 30 s over several hours. Then the coverslip was removed from the dish, plunged into fixative and stained with a fluorescent lectin for examination by confocal microscopy (see below). Fiji software was used to analyze the image sequences and to create a projection of the complete image stack, which revealed the entire track of the animal and allowed comparison with the mucous track observed by fluorescence microscopy. The binding rate of Alexa 647-WGA to mucus was assessed by time lapse imaging in a separate experiment.

### Co-labeling for mucus and endomorphin by light microscopy

For mucus labeling, samples were fixed in a mixture of paraformaldehyde (4%, EMS, Hatfield, PA, USA) and glutaraldehyde (0.25%; EMS, Hatfield, PA, USA) in buffered ASW (NaCl 400 mM; MgCl_2_ 5 mM; CaCl_2_ 2 mM; sucrose 300 mM; HEPES 30 mM; pH 7.4) for 30 min to visualize mucous trails, or for 2 h to visualize whole animals. To find a specific marker for *Trichoplax* mucus, several fluorescent lectins were tested: concanavalin A, *Dolichos biflorus* agglutinin, peanut agglutinin, *R. communis* agglutinin, soy bean agglutinin, *U. europaeus* agglutinin, and wheat germ agglutinin (Vector Laboratories, Burlingame, CA, USA). Lectins were diluted 1:200 in PBS for trail labeling or in PBS with 0.05% Triton (Sigma-Aldrich, St Louis, MO, USA) for tissue labeling. Hoechst dye (1:2000; Life Technologies, Carlsbad, CA, USA) was used to label nuclei. Samples were mounted in Vectashield medium (Vector Laboratories, Burlingame, CA, USA) and examined on a LSM 510 microscope (Carl Zeiss Microscopy, Jena, Germany).

For immunofluorescence, animals were frozen and freeze substituted as described previously (Smith et al., 2014) or fixed chemically as described above. Antibodies used were primary rabbit anti-endomorphin (1:800 in BB, Phoenix Pharmaceuticals, Burlingame, CA, USA) and secondary ATTO 488 goat anti-rabbit (1:500, Sigma-Aldrich, St Louis, MO, USA). WGA conjugated Alexa 555 (1:200, Thermo Fisher Scientific/Invitrogen, Eugene, OR, USA) was applied together with the secondary antibodies. Samples were mounted in Vectashield medium and examined with a LSM 880 with an AiryScan detector or LSM 800 (Carl Zeiss Microscopy, Jena, Germany). Colocalization of anti-endomorphin and WGA staining was evaluated with Zen software and Volocity image analysis software (Quorem Technologies, Inc. Puslinch, Ontario, Canada).

### Gland cell number estimation

The estimation was based on both SEM and LSM. The relative proportions of the three types of gland cells were determined by counting cells in tiled images of entire animals obtained by SEM (see above). We used the following formula to make a rough estimation of a total number of gland cells of each type in one animal:

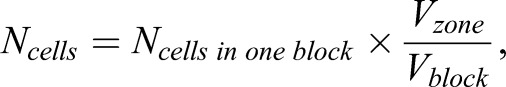
where *V_zone_* is the volume of the zone occupied by a cell of this type; *V_block_* is a volume of a block with thickness equal to a gland cell diameter and length equal to the width of the zone; *N_cells in one block_* is an average number of cells of this type averaged from seven tilled images. The proportions were 2:6:1 for Type 1:Type 2:Type 3. The total number of Type 2 cells (∼900 per 600 µm animal) was obtained by counting all WGA stained cells in a wholemount and the total number of Type 1 cells (∼280 per 600 µm animal) by counting all cells containing dark granules visible by transmitted light. The total number of Type 3 cells was estimated on the basis of their proportion relative to Type 1 and Type 2 cells.

### Fluorescence *in situ* hybridization

*In situ* hybridization experiments were performed with probes and reagents from Advanced Cell Diagnostics (Hayward, CA, USA). Animals were transferred to a 200 µl drop of ASW mixed an equal part 0.97 M mannitol (in water) on Superfrost Plus Gold glass slides (Thermo Fisher Scientific, Pittsburgh, PA, USA). After 2 h, the ASW/mannitol was removed and the slides were plunged into tetrahydrofuran on dry ice and kept overnight. The slides were transferred to 3% acetic acid in methanol at −20°C for 30 min followed by a mixture of 16% paraformaldehyde and methanol (1:3), initially at −20°C and then room temperature (RT) for 30 min. The samples were rinsed twice in methanol, dried for 5 min and then treated with Protease IV for 30 min at RT. Hybridization was performed with RNAscope Multiplex Fluorescent assay according to supplier instructions using the following RNAscope probes: Ta-Triaddraft_63128 (# 561031; trypsin), Ta-Triaddraft_57870 (#561141-C2; phospholipase A2), Ta-Phopholipase A2 (#572841) and Ta-Chymotrypsin (#572831-C2). Samples were mounted in ProLong™ Gold antifade reagent (Invitrogen, Eugene, OR, USA) and examined in Zeiss LSM 800 or LSM 880. Dissociated cells prepared from animals treated in 0.25% trypsin in calcium-free ASW for 45 min were plated in polylysine coated MatTek chambers (Ashland, MA, USA) with grid cover glasses. The chambers were processed as above except that they were frozen in methanol rather than tetrahydrofuran, fixed in MeOH with 4% paraformaldehyde and rehydrated prior to protease treatment (Protease III diluted 1:15) for 13 min. *In vivo* staining of lipophils with LysoTracker Red and LipidTox (Life Technologies, Carlsbad, CA, US) was performed as described (Smith et al., 2014). Fluorescence imaging was performed as described above.

## Supplementary Material

Supplementary information
