# Supplementary material for: The ventral epithelium of Trichoplax adhaerens deploys in distinct patterns cells that secrete digestive enzymes, mucus or diverse neuropeptides
Source: Biol Open. 2019 Jul 31;8(8):bio045674. doi: 10.1242/bio.045674 (PMC6737977; doi:10.1242/bio.045674)
Supplement: Supplementary information [file biolopen-8-045674-s1.pdf]

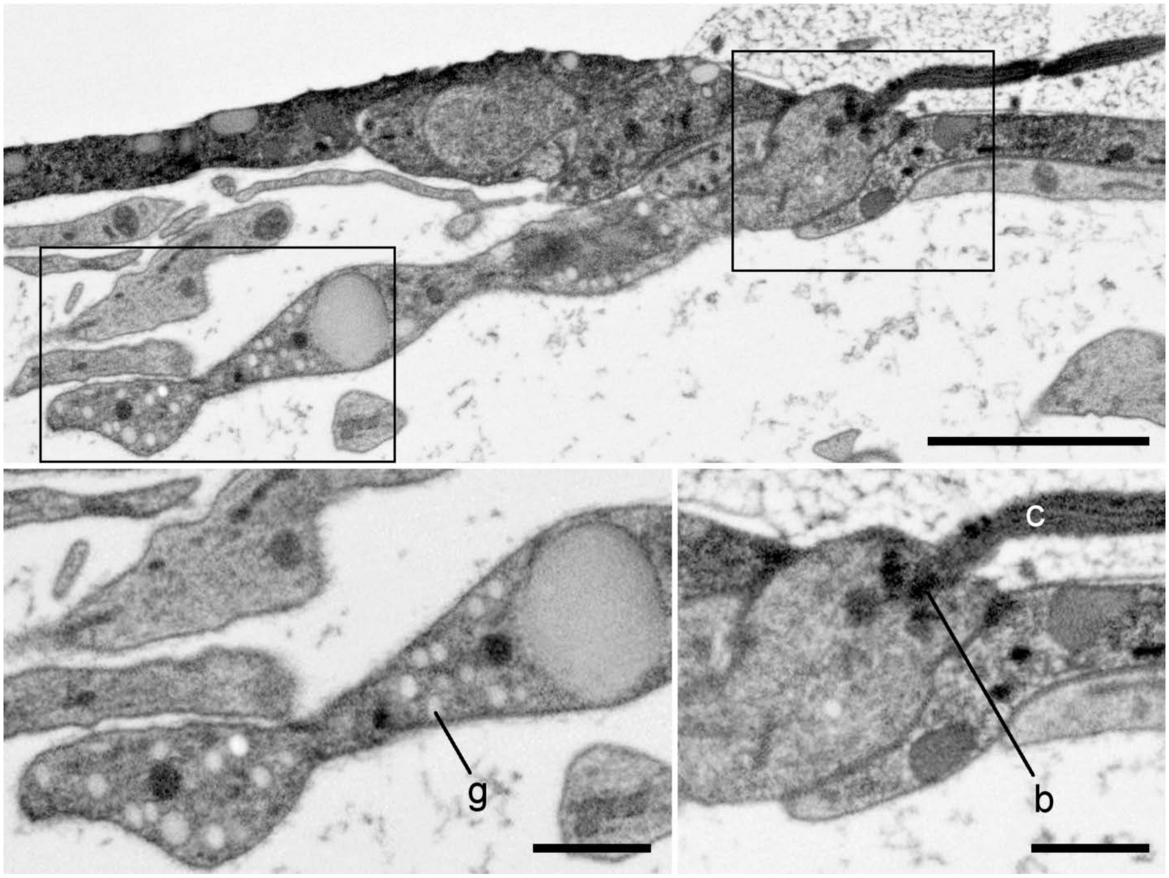


**Fig. S1**. Type 3 gland cell in the dorsal epithelium. Boxed areas are magnified in insets: Lower left inset shows the basal part of the cell body filled with granules (g) and lower right inset shows the cell apex with a cilium (c) and basal body (b). Scale bar 2 µm.

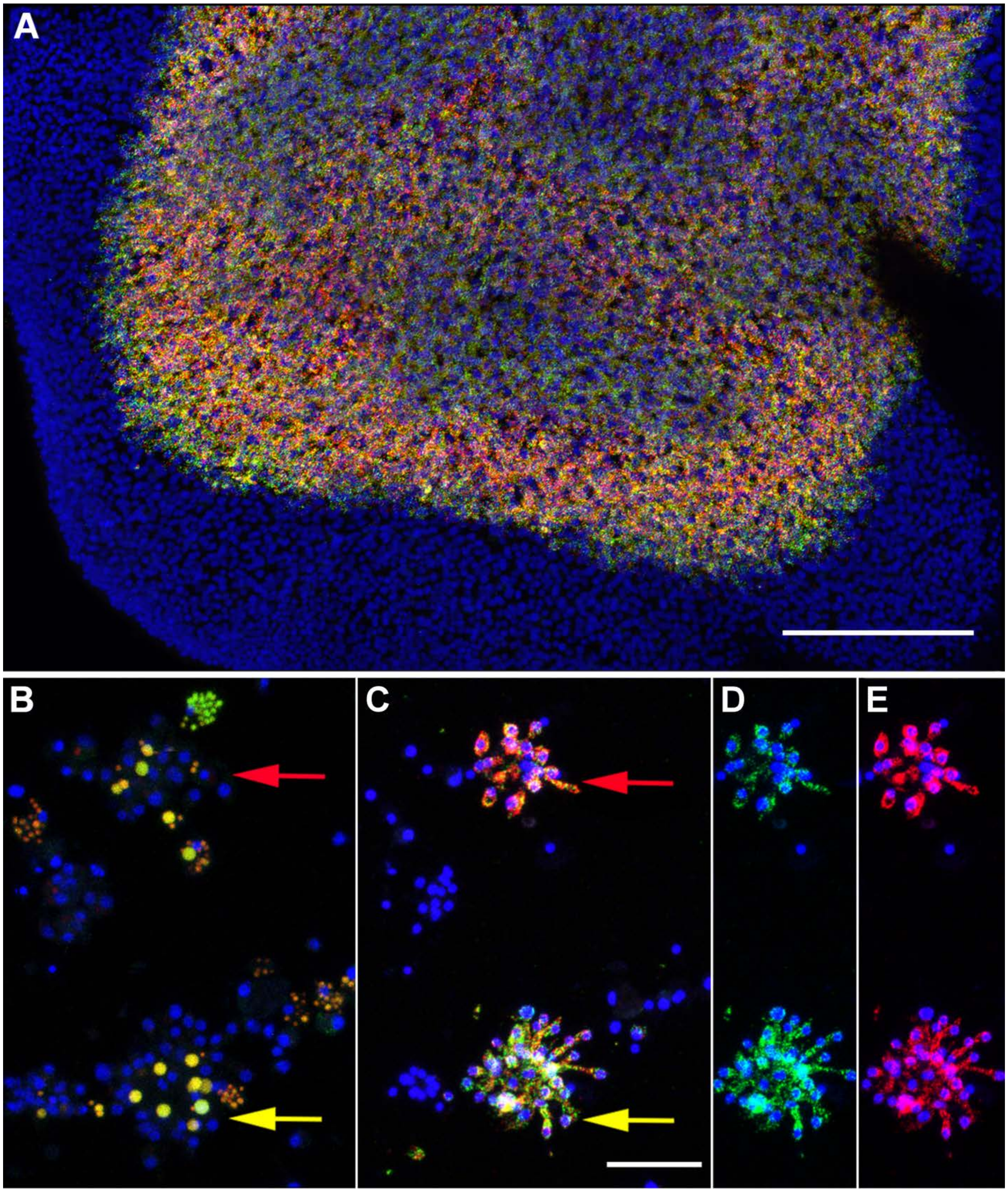


**Fig. S2**. FISH for phospholipase A2 (green) and chymotrypsin (red). Nuclear staining (Hoechst) is blue. **(A)** Maximum intensity projection of optical sections through an entire animal. **(B)** Dissociated cells labeled with lysotracker (red) and lipidTox (green) to mark lipophil cell granules prior to fixation. **(C)** Same field after fixation and hybridization with probes for phospholipase A2 and chymotrypsin. Red and yellow arrows indicate cell clusters that included lipophil granules **(B)** and were labeled by the probes **(C)**. **(D, E)** Color separated images of cell clusters in **(C)**. Scale bars: A – 50 µm, B-E – 20 µm.

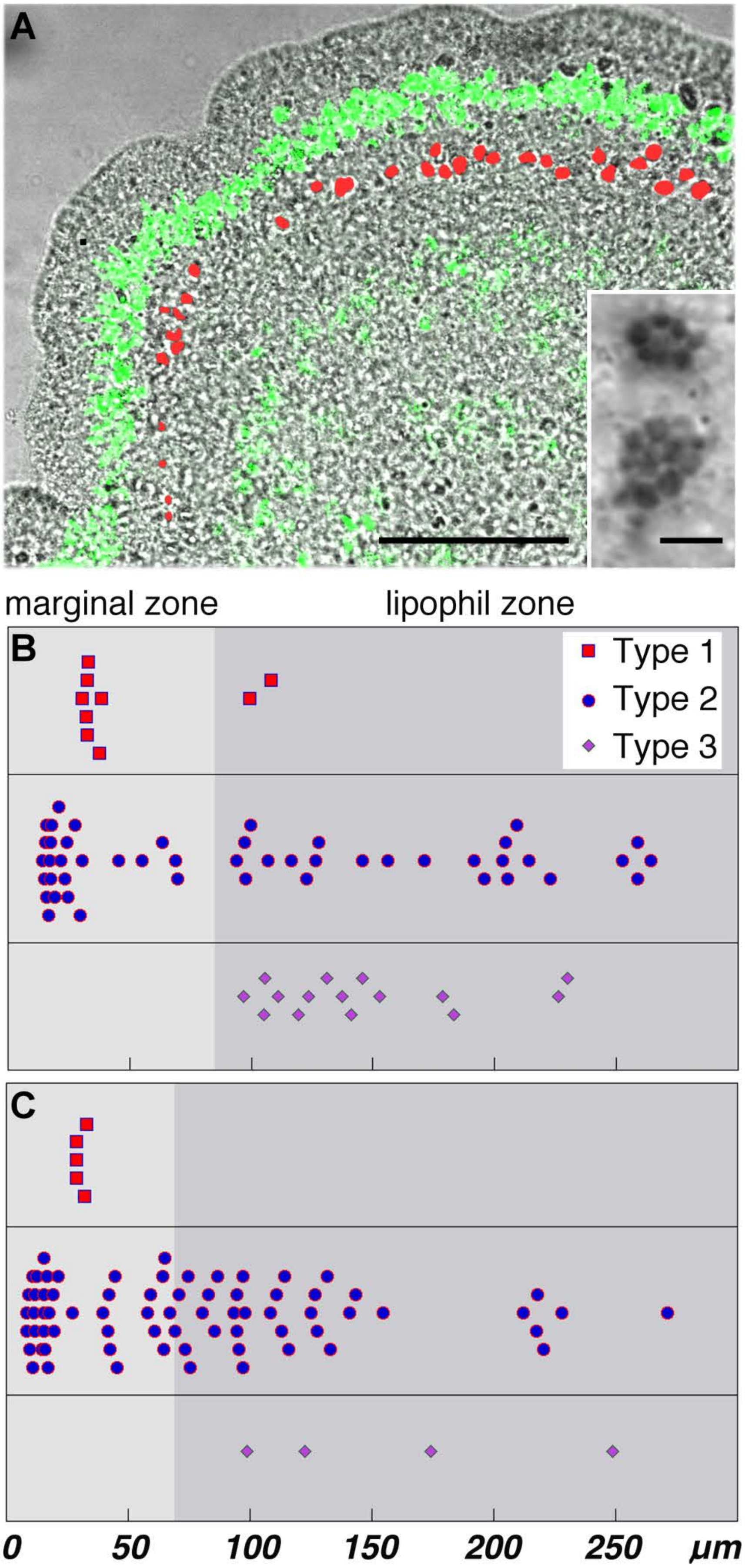
A
marginal zone
lipophil zone
B
Type 1
Type 2
Type 3
C
0
50
100
150
200
250
µm

**Fig. S3**. Distribution of gland cells. **(A)** WGA staining (green, projection of 15 sections) superimposed onto a single optical section in transmitted light showing a quadrant of *Trichoplax* body. Type 2 cells are visualized with WGA and Type 1 cells are artificially colored in red. Inset shows magnified view on two Type 1 cells with granules visible in transmitted light. **(B, C)** Graphical representations of the measured distributions of the three types of gland cells in two animals, 2 (B) and 3 (C). Measurements done in same way as for animal 1 shown in Figure 2I. Scale bars: A – 50 µm, inset on A – 3 µm.

Biology Open • Supplementary information

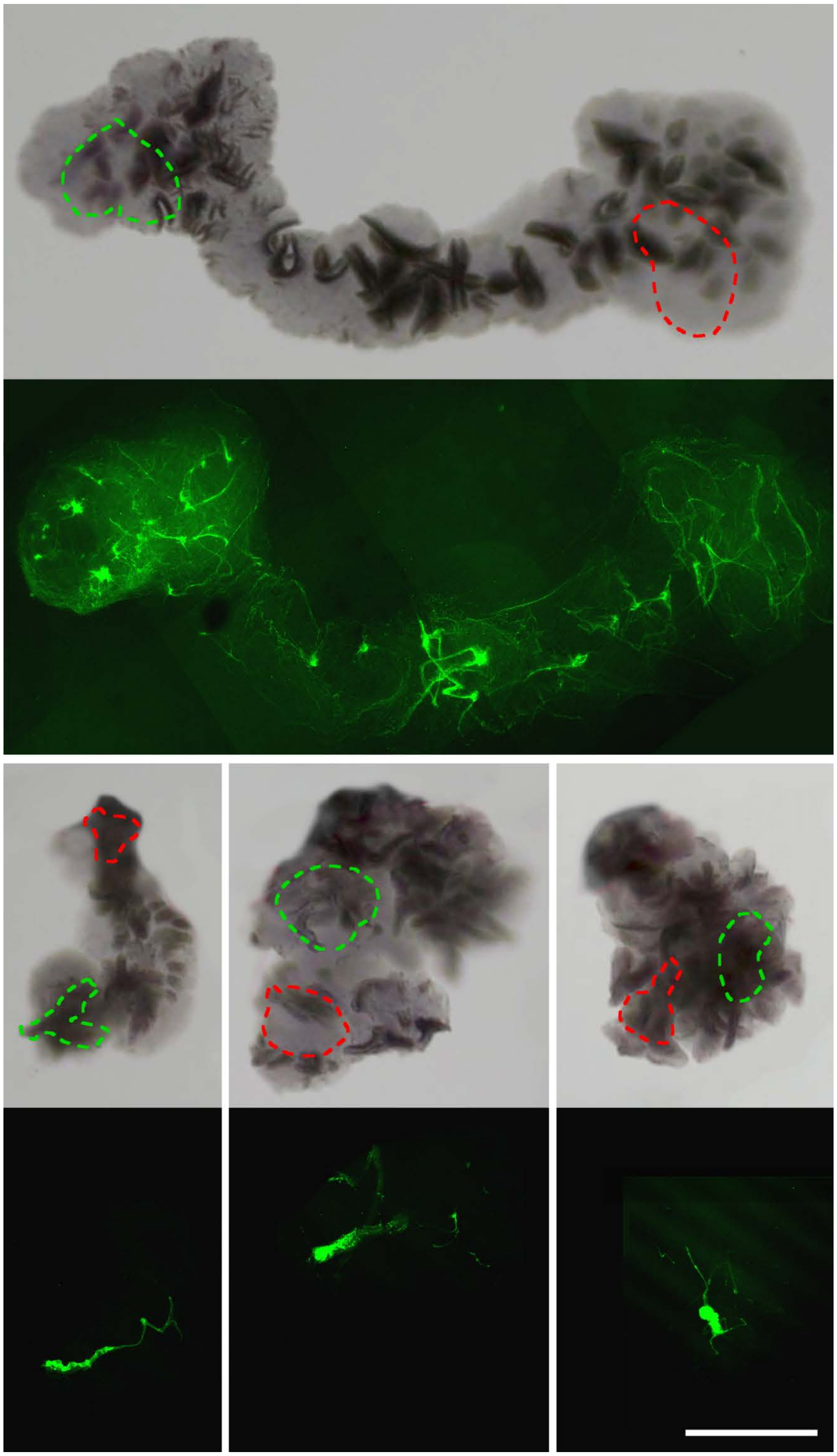

**Fig. S4**. Movements and mucous trails of animals in normal seawater (upper image) and calcium free seawater (lower images). Upper insets are projections of time lapse bright field image sequences (2 hour duration). Green and red dotted lines outline the perimeter of the animal in the first and last frames, respectively. Lower insets are respective mucous trails stained with fluorescent WGA (green). Scale bar 500 µm.

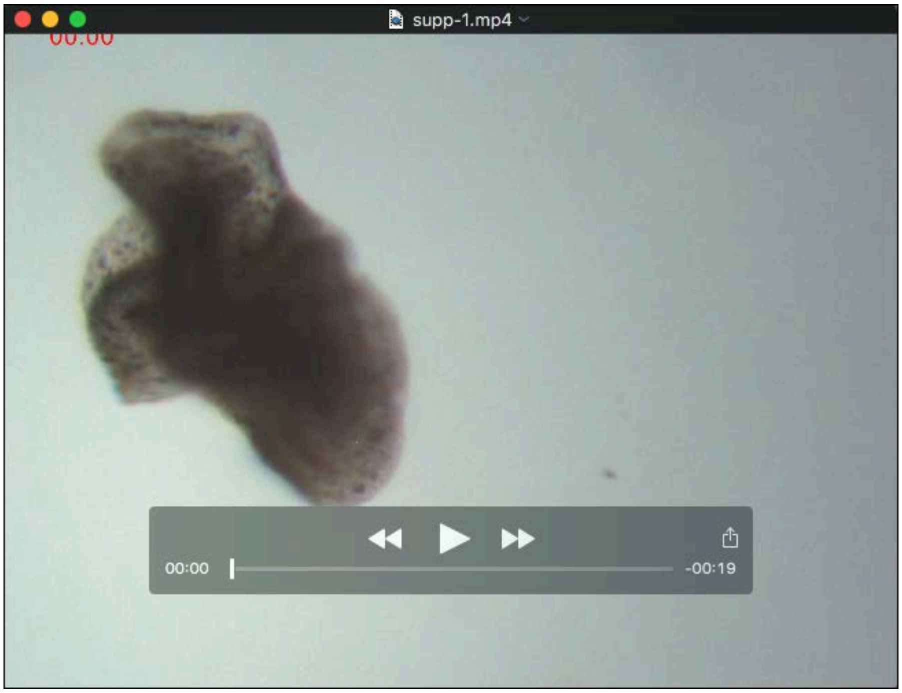


**Movie 1**. Attachment of *Trichoplax* to a substrate, top view. Time lapse recording one frame per 5 seconds. The animal folds up with the ventral surface inside after detachment from the substrate as seen at the beginning of the movie. It moves in place by ciliary beating. Then it unfolds slowly, establishing the contact with a substrate with an exposed surface of the ventral epithelium. The area of the contact with a substrate expands as the body spreads.
